# Secular Trends of Clinical Characteristics and Survival of Hepatocellular Carcinoma in Taiwan from 2011 to 2019

**DOI:** 10.3390/v15010126

**Published:** 2022-12-31

**Authors:** Kwong-Ming Kee, Chien-Hung Chen, Jui-Ting Hu, Yi-Hsiang Huang, Tsang-En Wang, Gar-Yang Chau, Kuo-Hsin Chen, Yao-Li Chen, Chih-Che Lin, Chien-Fu Hung, Shiu-Feng Huang, Tsang-Wu Liu, Hsiu-Ying Ku, Bing-Shen Huang, Yi-Pin Wang, Hui-Ping Tseng, Chun-Ju Chiang, Sheng-Nan Lu

**Affiliations:** 1 Graduate Institute of Clinical Medical Sciences, College of Medicine, Chang Gung University, Taoyuan City 83302, Taiwan; 2 Division of Hepato-Gastroenterology, Department of Internal Medicine, Kaohsiung Chang Gung Memorial Hospital and Chang Gung University College of Medicine, Kaohsiung City 83301, Taiwan; 3 Department of Internal Medicine, National Taiwan University Hospital Yunlin Branch, Douliu City 64041, Taiwan; 4 Department of Internal Medicine, National Taiwan University Hospital and National Taiwan University College of Medicine, Taipei City 10002, Taiwan; 5 Liver Unit, Cathay General Hospital, Taipei City 10630, Taiwan; 6 Division of Gastroenterology and Hepatology, Taipei Veterans General Hospital, Taipei City 11217, Taiwan; 7 Institute of Clinical Medicine, National Yang Ming Chiao Tung University School of Medicine, Taipei City 112, Taiwan; 8 Division of Gastroenterology, Department of Internal Medicine, MacKay Memorial Hospital, Taipei City 10449, Taiwan; 9 Division of General Surgery, Department of Surgery, Taipei Veterans General Hospital, Taipei City 11217, Taiwan; 10 Division of General Surgery, Department of Surgery, Far Eastern Memorial Hospital, New Taipei City 22060, Taiwan; 11 Department of Surgery, Changhua Christian Hospital, Changhua 50046, Taiwan; 12 Department of Surgery, Kaohsiung Chang Gung Memorial Hospital, Chang Gung University College of Medicine, Kaohsiung City 83301, Taiwan; 13 Department of Radiology, New Taipei Municipal Tucheng Hospital, New Taipei City 23652, Taiwan; 14 National Institute of Cancer Research, National Health Research Institutes, Miaoli 35053, Taiwan; 15 Department of Radiation Oncology, Chang Gung Memorial Hospital and Chang Gung University, Taoyuan City 33305, Taiwan; 16 Cancer Administration and Coordination Center, National Taiwan University Hospital, Taipei City 10002, Taiwan; 17 Cancer Center, Kaohsiung Chang Gung Memorial Hospital, Kaohsiung City 83301, Taiwan; 18 Graduate Institute of Epidemiology and Preventive Medicine, College of Public Health, National Taiwan University, Taipei 10002, Taiwan

**Keywords:** hepatitis C virus, hepatitis B virus, hepatocellular carcinoma, gender, age, non-B and non-C viral hepatitis, survival

## Abstract

Hepatocellular carcinoma (HCC) is a major cause of cancer death in Taiwan, and in the past 30–40 years, Taiwan has been committed to its prevention and treatment. We aimed to investigate the secular trends of characteristics and the survival of HCC in recent decades after making increased efforts. Between 2011 and 2019, a total of 73,817 cases were enrolled from the TCR database. The overall male-to-female ratio was 7/3. The overall, male and female mean ages increased from 63.8 to 66.1 years, 62.0 to 64.3 years and 68.3 to 70.4 years, respectively. After dividing by viral etiologies and gender, the mean age showed increasing trends in all subgroups. The proportions of HBV-HCC, HCV-HCC, HBV+HCV-HCC and Non-HBV+non-HCV-HCC were 48.3%, 25.2%, 5.3% and 21.3% in males, compared with 25.5%, 48.6%, 5.3% and 20.5% in females, respectively. The 5-year survival rates of BCLC stages 0, A, B, C and D were 70%, 58%, 34%, 11% and 4%, respectively. The proportion of BCLC stage 0 increased from 6.2% to 11.3%. Multivariate analysis showed that being female, older age, diagnostic year, BCLC stages, hospital level, body mass index, smoking, alcohol consumption, AFP, Child–Pugh classification and HBV/HCV status were independent predictors for survival. In recent decades, the overall survival of HCC in Taiwan has been improving and might be partly associated with increased BCLC 0 and Child–Pugh A patients, while with the consequent age of patients increasing over time. The proportion of viral-related HCC is decreasing, while nonviral-related HCC is increasing.

## 1. Introduction

Primary liver cancer was the sixth most common cancer and the third leading cause of cancer death worldwide in 2020 [1]. Hepatitis B virus (HBV), hepatitis C virus (HCV), alcohol consumption and liver cirrhosis patients are high-risk groups for HCC. In addition, the incidence of non-HBV and non-HCV-related HCC (NBNC-HCC) has also been increasing globally [2,3].

Hepatocellular carcinoma (HCC) has been one of the leading causes of cancer death in Taiwan, although the mortality rate has declined from first place in the past period to second place since 2010 [4]. Chronic HBV and HCV infections are endemic and major causes of HCC in Taiwan. In the past period, HBV carriers accounted for approximately 20% of the general population in Taiwan and increased the risk of HCC incidence [5]. The overall prevalence of HCV was 1.8∼5.5% in Taiwan, which is higher than the global prevalence [6,7]. Geographic aggregation patterns in HCV transmission have been observed in some hyperendemic areas for HCV infection [8].

Previous studies showed that HCC was a male-predominant cancer at younger ages, especially in patients with chronic HBV infection, while HCV infection was female-predominant with generally older age [1,2,9,10,11]. The improvement in instrumentation for tumor detection and treatment has improved HCC survival in recent decades.

HCC has been one of the most common cancers in Taiwan since the past four decades, although the ranking of liver cancer in Taiwan declined from first place for males in 1998–2002 to fourth place in 2019, while the ranking for females declines from third place in 1998–2002 to sixth place in 2019 [4]. The secular trend of clinical characteristics, such as age, gender and virological etiologies, and the overall survival of HCC in recent decades were previously not well understood in Taiwan, and it required a national database to clarify this issue. The aims of this study were to investigate the secular trends of clinical characteristics, such as age, sex, viral etiologies, tumor marker and Barcelona Clinic Liver Cancer (BCLC) status, and the survival of HCC in recent decades in Taiwan based on a nationwide database.

## 2. Materials and Methods

### 2.1. Patients and Methods

We obtained data of HCC (ICD-9 code = 155 and ICD-10 code = C22) patients from the Taiwan Cancer Registry (TCR) database [12]. The TCR is an accumulative nationwide population-based registry established in 1979 that collects the data of patients with newly diagnosed cancer from hospitals with 50 or more beds, providing high-quality completeness. This registry has established a long-form database that has recorded cancer staging, detailed treatment and information of tumor recurrence since 2002 [13], and up to 114 items have been recorded since 2011. Additionally, detailed information regarding HCC site-specific factors (SSF) has been collected since 2011, including serum alpha fetoprotein (AFP), creatinine, total bilirubin, prothrombin time, hepatitis B surface antigen (HBsAg), antibody to HCV (anti-HCV), Child–Pugh classification, presence of liver cirrhosis and BCLC staging system. BCLC stages have been registered since 2007 and became a formal item in 2011. Consequently, this study enrolled HCC patients for the period from 2011 to 2019, as more clinical information could be collected and analyzed from these time periods.

Relevant demographics collected in this study included age, gender, HBsAg, anti-HCV, AFP, Child–Pugh classification, history of smoking, alcohol consumption and BCLC stages. Viral hepatitis status was classified as HBV-related HCC (B-HCC), HCV-related HCC (C-HCC), HBV plus HCV-related HCC (B+C-HCC) and non-HBV- and non-HCV-related HCC (NBNC-HCC).

Secular trends of sex ratio, gender, age, BCLC stages, overall survival and associated factors were analyzed. The interval delay around 2 to 3 years for TCR after HCC patients was diagnosed; survival status was obtained from the national mortality database, while HCC survival was calculated and analyzed by linking and merging these two databases.

### 2.2. Statistical Analysis

Demographic and clinical variables were reported as a percentage or mean ± standard deviation, with categorical variables being compared using the Chi-squared test, and continuous variables were compared using Student’s *t*-test. Univariate analysis was conducted for all variables using the Cox proportional hazards model, and potential prognostic factors in the univariate analysis were chosen for multivariate analysis with respect to overall survival to assess the independent prognostic factors. Hazard ratios (HRs) with 95% confidence intervals (CIs) were analyzed. Long-term secular trends of individual factors were tested.

## 3. Results

### 3.1. Secular Trends of Sex Ratio and Age

**Figure 1:**
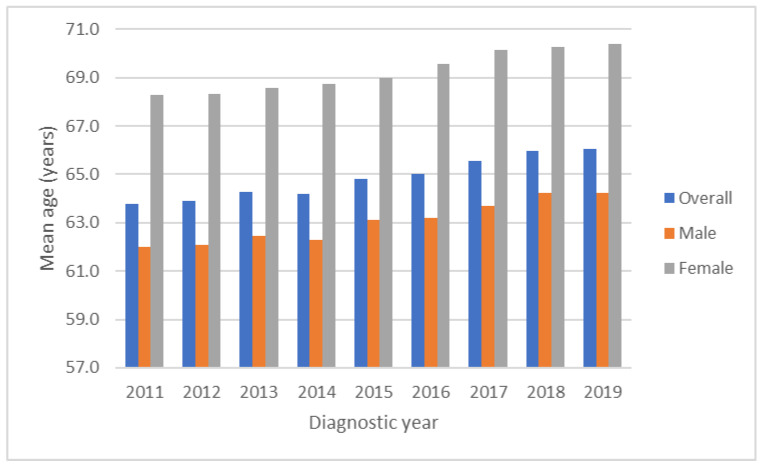
Secular trends of mean age from 2011 to 2019. The overall, male and female mean ages at diagnosis increasing annually over time (all *p* values for linear trend <0.01). Mean age of females were about 6 years older than males on average when diagnosis.

A total of 73,817 HCC cases identified between 2011 and 2019 were enrolled from the TCR database, including 52,437 males and 21,380 females. Figure 1 shows the secular trends of age from 2011 to 2019, with mean age of females being about 6 years older than males on average when diagnosed. The overall and mean ages for males and females at diagnosis significantly increased annually during the study period from 63.8 to 66.1 years, separately from 62.0 to 64.3 years and from 68.3 to 70.4 years, respectively (all *p* values for linear trend <0.01).

### 3.2. Secular Trends of Child–Pugh Classification

**Figure 2:**
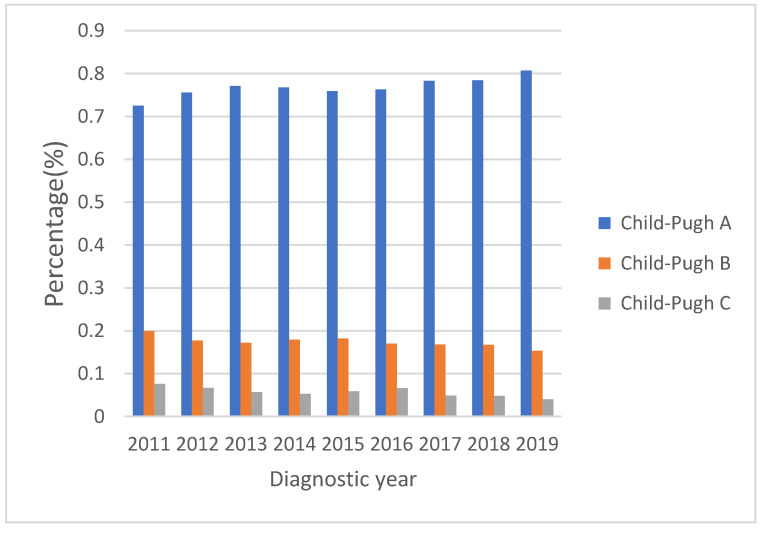
Child–Pugh classification from 2011 to 2019 shows liver function reserve improved over time (all *p* values for linear trend <0.01). Proportion of Child–Pugh class A increased from 72.5% to 80.7%. The proportions of Child–Pugh B and C decreased from 19.9% to 15.3% and from 7.6% to 4.0%, respectively.

Figure 2 shows the secular trends of the Child–Pugh classification with the proportion of Child–Pugh class A increasing from 72.5% to 80.7% and the proportions of Child–Pugh B and C decreasing from 19.9% to 15.3% and from 7.6% to 4.0%, respectively (all *p* values for linear trend <0.01). If we divided the study period into 2011–2014 and 2015–2019, the proportion of Child–Pugh class A increased from 75.6% to 77.9%, and the proportions of Child–Pugh B and C decreased from 18.1% to 16.8% and from 6.6% to 5.3%, respectively (all *p* values < 0.05).

### 3.3. Secular Trends of Viral Etiology Based on Gender

**Figure 3:**
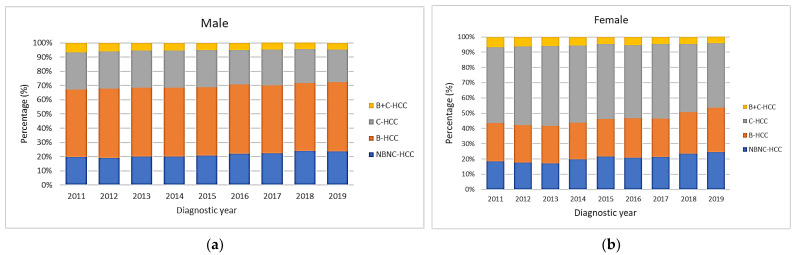
Secular trends of viral etiologies according to gender. (**a**) In male gender, HBV accounts for half of the cases. The proportion of NBNC-HCC increased over time, while the proportion of C-HCC decreased (all *p* values for linear trend <0.001). (**b**) In female gender, HCV accounts for nearly half of HCC cases. The proportions of B-HCC and NBNC-HCC increased, while the proportion of C-HCC decreased over time (all *p* values for linear trend <0.001).

The proportions of B-HCC, C-HCC, B+C-HCC and NBNC-HCC were 48.3%, 25.2%, 5.3% and 21.3% in males, compared with 25.5%, 48.6%, 5.3% and 20.5% in females, respectively. Figure 3a shows the secular trends of different viral etiologies based on the male gender and HBV-HCC accounting for half of HCC cases. The proportion of NBNC-HCC cases increased from 19.8% to 23.8%, while the proportion of C-HCC cases decreased from 26.2% to 23.1% significantly (all *p* values for linear trend <0.001). If divided into two periods with 2011–2014 and 2015–2019, the proportion of NBNC-HCC cases increased from 19.7% to 22.5%, while the proportions of C-HCC and B+C-HCC cases decreased from 26.2% to 24.5% and from 5.9% to 4.8%, respectively (all *p* values < 0.05). Figure 3b shows the secular trends of different viral etiologies for the female gender with HCV-HCC accounting for nearly half of HCC cases. The proportion of B-HCC increased from 25.0% to 29.1%, and NBNC-HCC also increased from 18.5% to 24.4% significantly, while the proportion of C-HCC decreased from 49.7% to 42.4% significantly (all *p* values for linear trend <0.001). If divided into two periods with 2011–2014 and 2015–2019, the proportions of B-HCC and NBNC-HCC increased from 24.6% to 26.3% and from 18.2% to 22.3%, respectively. The proportions of C-HCC and B+C-HCC decreased from 51% to 46.7% and from 6.2% to 4.7%, respectively (all *p* values < 0.05).

### 3.4. Secular Trends of Age Based on Viral Etiology and Gender

**Figure 4:**
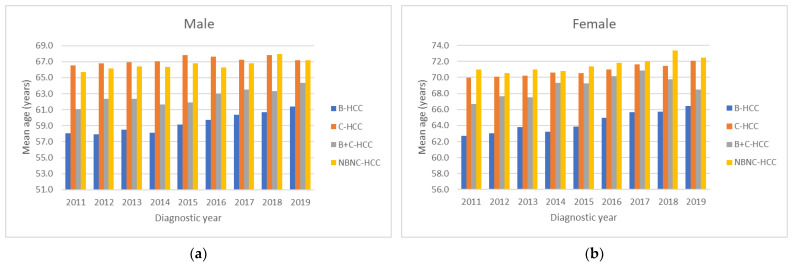
All mean ages during the time period classified according to gender and viral etiology showed increasing trends in both male (**a**) and female (**b**) gender (all *p* values for linear trend <0.01).

Figure 4 shows the mean age for HCC diagnosis according to different viral etiologies. The trends of mean age all increased in both males (Figure 4a) and females (Figure 4b) significantly (all *p* values for linear trend <0.01). The youngest age was in male HBV-HCC cases in 2011 (58.0 years), while the oldest age was in female HCV-HCC cases in 2018 (73.4 years). For the male gender (Figure 4a), the mean age of B-HCC patients (59.3 years) was younger than that of B+C-HCC (62.6 years), NBNC-HCC (66.6 years) and C-HCC (67.2 years) patients, while for the female gender (Figure 4b), the mean age of B-HCC patients (64.3 years) was younger than that of B+C-HCC (68.9 years), C-HCC (70.9 years) and NBNC-HCC (71.6 years) patients.

### 3.5. Secular Trends of Male-to-Female Ratio Based on Viral Etiology

**Figure 5:**
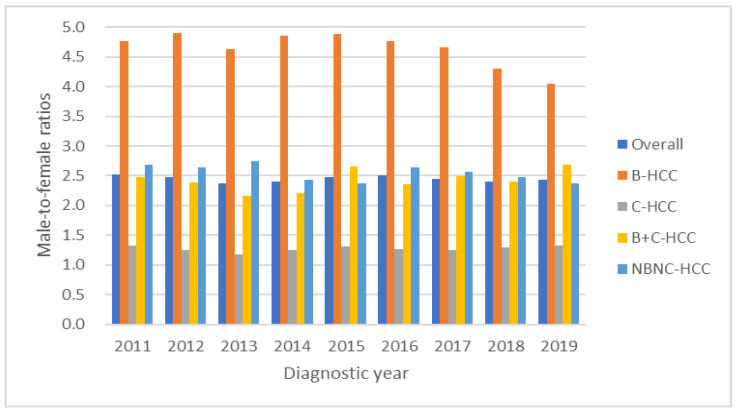
The male-to-female ratios of overall, B-HCC, C-HCC, B+C-HCC and NBNC-HCC were 2.4, 4.6, 1.3, 2.4 and 2.5, respectively. The B-HCC showed decreasing trend from 4.8 to 4.1 during the study period.

The overall male-to-female ratio was 2.4. The secular trends of male-to-female ratios were 2.5, 2.5, 2.4, 2.4, 2.5, 2.5, 2.4, 2.4 and 2.4 (*p* values for linear trend = 0.82) from 2011 to 2019, with these results showing no significant change during the study period. With male-to-female ratios divided based on viral etiology (Figure 5), the overall male-to-female ratios of B-HCC, C-HCC, B+C-HCC and NBNC-HCC were 4.6, 1.3, 2.4 and 2.5, respectively. B-HCC showed decreasing significance from 4.8 to 4.1 during the study period (all *p* for linear trend <0.01); otherwise, C-HCC, B+C-HCC and NBNC-HCC showed no significant change over time.

### 3.6. Secular Trends of BCLC Stages

**Figure 6:**
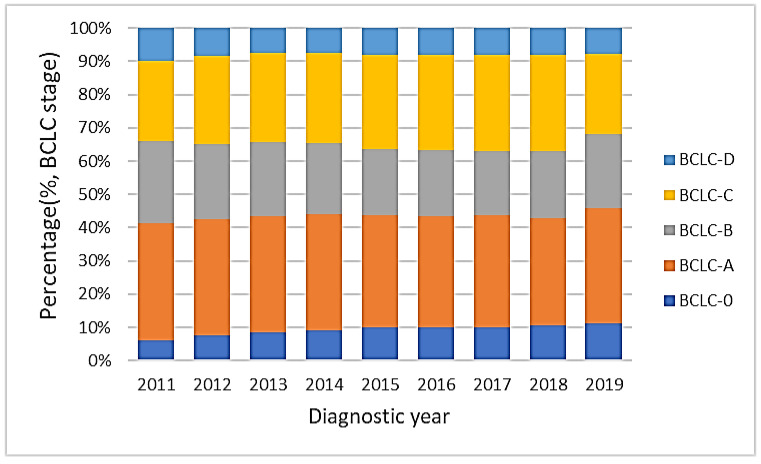
The distribution of BCLC stages from 2011 to 2019. The proportion of BCLC stage 0 increased from 6.2% to 11.3% (*p* value for linear trend <0.01), while the proportion of both BCLC stages A and B decreased (*p* values for linear trend <0.01). The sum of BCLC stages 0, A and B did not change significantly.

Figure 6 shows the distribution of BCLC stages 0, A, B, C and D from 2011 to 2019. The proportion of BCLC stage 0 increased from 6.2% to 11.3% significantly (*p* value for linear trend <0.01), while the proportions of both BCLC stages A and B decreased over time (*p* values for linear trend <0.01). BCLC stages C and D and the sum of BCLC stages 0, A and B did not differ significantly.

### 3.7. Overall Survival and Associated Prognostic Factors

The overall survival rates of HCC patients showed significant differences in each BCLC stage. The 5-year survival rates of BCLC stages 0, A, B, C and D were 70%, 58%, 34%, 11% and 4%, respectively. The median survival years of BCLC stages 0, A, B, C and D were 9.7, 6.3, 2.7, 0.6 and 0.2 years, respectively.

Table 1 shows the factors associated with HCC survival from 2011 to 2019. Multivariate analysis showed that gender, diagnostic age, diagnostic year, BCLC stages, hospital level, body mass index, smoking, alcohol consumption, serum AFP levels, Child–Pugh classifications and HBV and HCV status were all independent predictors for survival.

**Figure 7:**
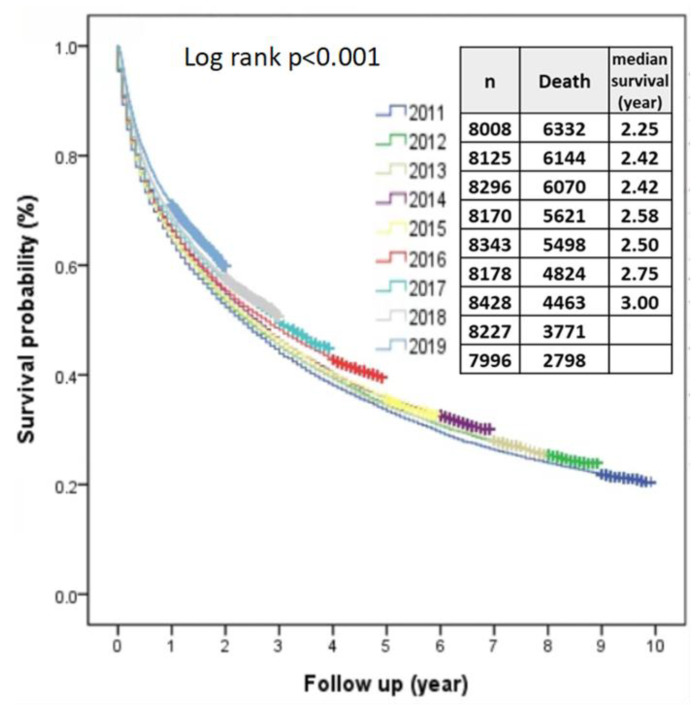
The overall median survival rates of HCC from 2011 to 2019 improved year-by-year.

Survival rate increased year-by-year from 2011 to 2019 significantly (*p* value for linear trend <0.001) (Figure 7). Using the year 2011 as a reference, in univariate analysis, hazard ratios (HR) gradually decreased to 0.768 (95% CI, 0.734–0.804; *p* < 0.001) in 2019, and after multiple adjustment, the HR in 2019 was 0.839 (95% CI, 0.800–0.8790; *p* < 0.001). The BCLC stage was another independent predictor, and compared to BCLC stage 0, HRs from BCLC stages A, B, C and D were 1.288 (95% CI, 1.226–1.352; *p* < 0.001), 2.296 (95% CI, 2.185–2.412; *p* < 0.001), 4.882 (95% CI, 4.65–5.125; *p* < 0.001) and 7.575 (95% CI, 7.124–8.056; *p* < 0.001), respectively.

## 4. Discussion

Our data were obtained from TCR, which is an accumulative nationwide population-based registry established in 1979 that collects the data of patients with newly diagnosed cancer and is one of the high-quality cancer registries globally [12,14], providing high-quality information for cancer surveillance and cancer incidence monitoring in Taiwan [15].

The major two causes of HCC in Taiwan are related to chronic HBV and HCV infections. The HBV nationwide vaccination program was launched in 1984, and the long-term effects of HBV vaccination have reduced the incidence of childhood-related HCC and HBV carrier rates in Taiwan [16]. Nucleos(t)ide analogues and interferon-based treatment have been covered under the National Health Insurance (NHI) program for chronic HBV and HCV patients since 2003, respectively. A nationwide study in Taiwan showed that the long-term effects of anti-viral therapies has decreased the incidence of B-HCC [17].

The proportion of C-HCC has also been decreasing in recent decades in Taiwan. The major cause could be the reimbursement of costs from the NHI scheme for nationwide interferon-based treatment since 2003 and successful anti-viral therapy treatment, thereby decreasing the incidence of HCC [18,19]. A piece of information concerning the effect of antiviral therapies on the results obtained from the C-HCC patient sample during the study period 2011–2019. A community-based study showed an overall estimation of a 4.4% HCV seroprevalence in Taiwan [7]; however, the prevalence of HCV of less than 1.4% among people born after 1960 with a decreasing trend in new HCV infections might be one of the major causes of the decreasing incidence of C-HCC in recent decades. The Ministry of Health and Welfare (MOHW) of Taiwan announced the “Taiwan Hepatitis C Policy Guidelines 2018–2025” in 2017 and financed the program to eliminate HCV infections for those patients with detectable viremia [20]. The high efficacy of direct-acting anti-viral agents in HCV treatment has played an important role in prevention and should further reduce the incidence of C-HCC in the future.

In recent decades, the incidence of non-viral-related HCC has increased worldwide [2,3,11,21,22]. A previous study from eight medical centers in Taiwan showed that the percentage of NBNC-HCC was around 10% between 1981 and 2001 [9]. In this study, the percentage of NBNC-HCC has increased up to around 20% in the recent period, demonstrating an increasing trend. The causes of NBNC-HCC might be associated with metabolic, environmental and genetic factors, as well as independent factors of alcohol consumption and smoking, all playing important roles in hepatocarcinogenesis, and patients with the latter two factors reveal poorer prognosis. With Taiwan being an endemic country for HBV, the proportions of spontaneous and treatment-related HBsAg seroclearance could play a role in NBNC-HCC, especially in patients with older age [23]. The actual number of HBsAg seroclearance cases is still unknown in NBNC-HCC, as serum anti-HBc antibody was not routinely examined and could not be obtained at nationwide levels. The identification of risk factors and enrollment into the surveillance programs are essential for these cases to achieve better HCC control [22].

The mean age in the current period (2011~2019) was significantly older if compared with a past period (1986~2001) [9] over time (64.8 vs. 57.9 years). In addition, the mean age in all subgroups divided by age and gender showed a similar increasing trend also being significantly higher than a previous study [9]. Although effective antiviral therapy has apparently reduced the incidences of B-HCC and C-HCC [16,17,18], the risk of HCC occurrence still exists among these patients, with the major risk factors being underlying liver cirrhosis and older age [24,25]; however, the onset of HCC might be delayed to an older age due to absence of viral activity after effective antiviral treatments. Additionally, older age had a positive correlation with the incidence rates of HCC, and the onset time tended to increase [11].

The male-to-female ratios reveal variation between different viral etiologies. The overall male-to-female ratios of B-HCC, C-HCC, B+C-HCC and NBNC-HCC were 4.6, 1.3, 2.4 and 2.5, respectively, although no significant changes in trend for C-HCC, B+C-HCC and NBNC-HCC were found. Compared with a previous study, the overall male-to-female ratio in B-HCC decreased from 6.4 in 1981~2001 to 4.6 in the current period [9]; additionally, the ratio showed a significant decreasing trend during this study period. The cause of decreased male-to-female ratios of B-HCC might be related to the large percentage decrease in female C-HCC. Furthermore, the onset age of HCC in females tended to be six years older than males, especially in B-HCC cases. Previous studies have shown that estrogen is associated with decreased IL-6-related viral replication and hepatic inflammation [10,26] and had protective effects in females [27]. Our study showed that there was a gender difference in HCC. Previous studies demonstrated that chronic HBV and HCV infection is male-dominant; HBV and HCV infection could enhance androgen receptor-mediated signaling that potentially triggers the carcinogenesis and progression of liver fibrosis [28,29,30,31,32,33].

The overall survival of HCC in recent decades has been improving in Taiwan. Compared with a previous period (1986–2002) in a medical center, the overall 5-year survival rates of BCLC stages 0 (70% vs. 47.9%), A (58% vs. 35%), B (34% vs. 18.2%), C (11% vs. 1.9%) and D (4% vs. 0.7%) were all significantly increased [34]. The median survival years of BCLC stages 0 (9.7 vs. 3.9), A (6.3 vs. 2.9), B (2.7 vs. 1.5), C (0.6 vs. 0.2) and D (0.2 vs. 0.1) were also increased. The major reasons are that the percentage of preserved liver function has increased along with the early detection and appropriate treatment of HCC [35,36]. The long-term benefit of successful viral control in HBV prevents hepatic decompensation, liver cirrhosis and HCC [37]. The viral eradication of HCV markedly decreases hepatic inflammation, further preserving liver function and the incidence of HCC [19,38]. The median survival years of BCLC stages 0, A and B are higher in the current study if compared with the BCLC treatment guidelines in 2022. The global HCC BRIDGE study showed higher survival rates of HCC patients in Taiwan, and this might be associated with higher rates of patients receiving tumor resection in the earlier stages of HCC [39]. Aggressive treatment options, such as tumor resection, are more common in Asian countries, including Taiwan, so survival rates should be higher in BCLC stages 0 and A patients, even in selected BCLC stages B or C HCC patients [34,40]. The expected median survival year of the current BCLC treatment guidelines in 2022 is higher than in BCLC stages C and D [41]. Current NHI reimbursement for first-line systemic treatments in patients with BCLC stage C is only available for targeted therapy with Sorafenib and Lenvatinib. The combination of atezolizumab with bevacizumab as a first-line treatment, with superior survival benefits compared to sorafenib, is not yet covered by the NHI in Taiwan [42,43]. Looking forward to the highly developed era of systemic therapies in Taiwan, further analysis can anticipate the effectiveness of systemic therapy in survival improvement in the future.

In Taiwan, combined serum AFP levels and ultrasonography have been used for surveillance in patients with chronic HBV and HCV infections in intervals of 3–6 and 6–12 months for cirrhosis and chronic liver disease, respectively [44,45]. The identification of a high-risk group for active surveillance with early detection has led to an increased proportion of BCLC stages migrating to BCLC 0 from BCLC A and B, although no significant changes in proportions in patients with BCLC stage C and D have been found.

Greater effort on the awareness of diseases and emphasis on the importance of HCC surveillance for high-risk groups is necessary to increase early detection rates and improve overall survival. The accessibility of HCC surveillance in remote areas should also be strengthened, where identifying high-risk patients and performing ultrasounds in such remote areas by primary care physicians are important for effective surveillance [46]. Currently, Taiwan is implementing remote ultrasound in health centers, and primary care physicians can connect in real-time with experts from medical centers that provide remote guidance in diagnosis and recommendation. If ultrasound is not available, some clinical methods, such as the GALAD score, are also an option for surveillance [47,48]. The nationwide HBV and HCV screening program for residents older than 45 was launched in September 2020, so more high-risk groups have been identified and enrolled in the surveillance program.

One major limitation of this study is the lack of detailed information and clinical characteristics concerning patient disease history, etiologies of NBNC-HCC such as history of diabetes, lipid profiles, fatty liver disease and information on antiviral therapies for HBV and HCV, so further studies are needed for clarification.

## 5. Conclusions

In summary, the overall survival rate of HCC in Taiwan has been improving, benefiting mainly from early tumor detection and effective antiviral treatment to reduce the incidence of HCC and preserve liver function. The proportion of gender, age and viral etiologies of HCC have been changing in Taiwan over time from 2011 to 2019, and with the percentage and importance of NBNC-HCC increasing, the necessary identification of these high-risk patients and routine surveillance has become easier.

## Figures and Tables

**Table 1:** Factors associated with survival of hepatocellular carcinoma from 2011 to 2019 in Taiwan.

		Univariate				Multivariate			
		HR	95% CI		*p* Value	HR	95% CI		*p* Value
Sex	Male	1				1			
	Female	0.901	0.882	0.919	<0.001	0.916	0.894	0.938	<0.001
Age (years)	<56	1				1			
	56–65	0.947	0.921	0.972	<0.001	1.127	1.096	1.158	<0.001
	66–75	1.075	1.046	1.104	<0.001	1.342	1.304	1.38	<0.001
	>75	1.629	1.585	1.674	<0.001	1.698	1.648	1.749	<0.001
Diagnostic year	2011	1				1			
	2012	0.954	0.921	0.988	0.0090	1.004	0.969	1.04	0.831
	2013	0.948	0.915	0.982	0.0030	0.999	0.964	1.036	0.961
	2014	0.912	0.88	0.946	<0.001	0.969	0.934	1.005	0.092
	2015	0.925	0.892	0.96	<0.001	0.953	0.918	0.989	0.012
	2016	0.867	0.835	0.901	<0.001	0.9	0.865	0.935	<0.001
	2017	0.842	0.81	0.876	<0.001	0.852	0.819	0.887	<0.001
	2018	0.828	0.794	0.862	<0.001	0.842	0.807	0.878	<0.001
	2019	0.768	0.734	0.804	<0.001	0.839	0.8	0.879	<0.001
BCLC stages	0	1				1			
	A	1.472	1.402	1.545	<0.001	1.288	1.226	1.352	<0.001
	B	2.962	2.821	3.11	<0.001	2.296	2.185	2.412	<0.001
	C	7.458	7.113	7.82	<0.001	4.882	4.65	5.125	<0.001
	D	16.235	15.408	17.106	<0.001	7.575	7.124	8.056	<0.001
Hospital level, n (%)	Medical center	1				1			
	Regional hospital	1.268	1.245	1.292	<0.001	1.209	1.186	1.232	<0.001
Body mass index (kg/m^2^)	Normal (18.5~24)	1				1			
	Underweight (<18.5)	1.534	1.47	1.6	<0.001	1.176	1.127	1.227	<0.001
	Overweight (>24)	0.794	0.779	0.81	<0.001	0.895	0.877	0.913	<0.001
	Unknown	2.011	1.94	2.085	<0.001	1.177	1.134	1.223	<0.001
Smoking status	Non-smoker	1				1			
	Smoker	1.262	1.238	1.285	<0.001	1.18	1.155	1.206	<0.001
	Unknown	2.246	2.081	2.424	<0.001	1.162	1.074	1.257	<0.001
Alcohol consumption	No	1				1			
	Yes	1.056	1.035	1.078	<0.001	1.029	1.008	1.051	0.007
	Unknown	1.038	0.948	1.137	0.4200	1.125	1.027	1.233	0.011
Alpha Fetoprotein (ng/mL)	<20	1				1			
	20–399	1.448	1.414	1.483	<0.001	1.401	1.367	1.435	<0.001
	400–5999	2.284	2.223	2.347	<0.001	1.774	1.726	1.824	<0.001
	≥6000	3.383	3.298	3.469	<0.001	2.195	2.137	2.254	<0.001
Child–Pugh class	A	1				1			
	B and C	3.574	3.501	3.648	<0.001	2.022	1.924	2.124	<0.001
	Unknown	1.677	1.617	1.739	<0.001	1.33	1.278	1.384	<0.001
HBV and HCV status	Non-B–Non-C	1				1			
	Only HBV	0.723	0.705	0.74	<0.001	0.901	0.879	0.924	<0.001
	Only HCV	0.764	0.745	0.783	<0.001	0.897	0.874	0.921	<0.001
	HBV+HCV	0.806	0.771	0.842	<0.001	0.951	0.91	0.994	0.027
	Unknown	0.947	0.883	1.016	0.1300	0.742	0.687	0.802	<0.001

## Data Availability

The data that support the findings of this study are available from the corresponding author upon reasonable request.
